# Behavioral and neurophysiological correlates of emotional face processing in borderline personality disorder: are there differences between men and women?

**DOI:** 10.1007/s00406-022-01434-4

**Published:** 2022-06-05

**Authors:** Martin Andermann, Natalie A. Izurieta Hidalgo, André Rupp, Christian Schmahl, Sabine C. Herpertz, Katja Bertsch

**Affiliations:** 1 Department of Neurology, Heidelberg University Hospital, Heidelberg, Germany; 2 Department for General Psychiatry, Center of Psychosocial Medicine, Heidelberg University Hospital, Heidelberg, Germany; 3 School of Medicine, Universidad San Francisco de Quito, Quito, Pichincha Ecuador; 4 Department of Psychosomatic Medicine and Psychotherapy, Central Institute of Mental Health Mannheim, Medical Faculty Mannheim, Heidelberg University, Mannheim, Germany; 5 Department of Psychology, Ludwig-Maximilians-University Munich, Leopoldstr. 13, 80802 Munich, Germany; 6 NeuroImaging Core Unit Munich (NICUM), University Hospital LMU, Munich, Germany

**Keywords:** Borderline, Emotion, Facial expression, Sex, EEG, Source modeling

## Abstract

Emotional dysregulation is a core feature of borderline personality disorder (BPD); it is, for example, known to influence one’s ability to read other people’s facial expressions. We investigated behavioral and neurophysiological foundations of emotional face processing in individuals with BPD and in healthy controls, taking participants’ sex into account. 62 individuals with BPD (25 men, 37 women) and 49 healthy controls (20 men, 29 women) completed an emotion classification task with faces depicting blends of angry and happy expressions while the electroencephalogram was recorded. The cortical activity (late positive potential, P3/LPP) was evaluated using source modeling. Compared to healthy controls, individuals with BPD responded slower to happy but not to angry faces; further, they showed more anger ratings in happy but not in angry faces, especially in those with high ambiguity. Men had lower anger ratings than women and responded slower to angry but not happy faces. The P3/LPP was larger in healthy controls than in individuals with BPD, and larger in women than in men; moreover, women but not men produced enlarged P3/LPP responses to angry vs. happy faces. Sex did not interact with behavioral or P3/LPP-related differences between healthy controls and individuals with BPD. Together, BPD-related alterations in behavioral and P3/LPP correlates of emotional face processing exist in both men and women, supposedly without sex-related interactions. Results point to a general ‘negativity bias’ in women. Source modeling is well suited to investigate effects of participant and stimulus characteristics on the P3/LPP generators.

## Introduction

Borderline personality disorder (BPD) is a complex mental disorder characterized by severe impairments in self- and interpersonal functioning [1], including an unstable sense of self, hypersensitivity to interpersonal slights, volatile emotions, and impulsive behavior [2, 3]. Emotional dysregulation, i.e., the inability to flexibly respond to and manage emotions [4], is a core feature of BPD comprising an elevated ﻿emotional sensitivity, heightened and labile negative affect, deficits of appropriate regulation strategies, as well as a surplus of maladaptive regulation strategies [4].

A well-established way to assess emotion dysregulation is to consider how individuals with BPD respond to emotional stimuli, such as emotional facial expressions. Existing studies vary in their conceptual and methodological details [5], and results are inconsistent regarding, e.g., altered emotion recognition accuracy and sensitivity in individuals with BPD, or the question whether BPD-related differences are restricted to a subset of emotions [6–14]. There is, however, strong evidence that individuals with BPD show a negativity bias in response to faces, especially those with neutral or ambiguous valence [4, 15–19]. Such a biased social perception may be seen as an important mechanism for interpersonal dysfunctions, since it may hinder the individual to initiate social interactions and to form and maintain meaningful interpersonal relationships [20]. In fact, previous studies from our group have shown significantly more misclassifications of other emotional or neutral faces as angry, as well as more and faster initial eye movements to the eye region of angry faces [19, 21, 22], which were associated with self-reported aggression [19], a dysfunctional emotion regulation strategy. On the neural processing level, emotion dysregulation in individuals with BPD has been linked to functional imbalances within a fronto-limbic network, including amygdala hyperarousal and altered frontal activation in response to negative emotional facial expressions or scenes [15, 17, 18, 21, 23–34].

Most neuroscientific BPD studies have employed functional magnetic resonance imaging (fMRI); in contrast, electroencephalography (EEG) studies are rare, although EEG offers excellent capabilities to assess the spatio-temporal dynamics of cortical emotion processing [35, 36]. In particular, the centro-parietal activity after 300 ms [37, 38] is strongly modulated by the emotional significance of a stimulus [39, 40]; we will refer to this activity as P3/LPP (late positive potential), following Schindler and Bublatzky [36], to acknowledge the methodological and terminological diversity of previous research on this wave. Experiments in healthy participants have demonstrated that the P3/LPP magnitude is enhanced in response to emotionally intense, arousing, or salient as compared to neutral stimuli [39, 41–43]. Fearful or angry faces have been reported to elicit larger P3/LPP responses than faces with happy or neutral affect [36, 44, 45], suggesting that this wave might reflect a form of the above-mentioned negativity bias even in healthy individuals.

Given its role in emotion processing, the P3/LPP has received surprisingly little attention in BPD research. Existing studies have yielded mixed results with respect to overall P3/LPP magnitude differences between individuals with BPD and healthy controls [46–52]; however, these works must be considered with caution in the context of emotion processing since they employed oddball or gaming paradigms and analyzed P3/LPP sub-components from more frontal brain regions. Popkirov et al. [53] found no BPD-related effects in the P3/LPP responses to images with neutral and negative emotional valence; on the other hand, Marissen et al. [54] reported enhanced P3/LPP waves in individuals with BPD relative to healthy controls in stimuli with negative, but not positive or neutral valence. An investigation from our group [13], in turn, revealed smaller P3/LPP amplitudes in women with BPD, and this effect was pronounced in response to faces with happy expression; the finding was later extended to individuals with remitted BPD [55]. Interestingly, P3/LPP amplitudes were elevated in healthy controls in response to predominantly angry and happy faces, in line with a valence-independent elevated salience and arousal, while, consistent to the negativity bias, only predominantly angry but not happy faces elicited increased P3/LPP amplitudes in the BPD group. Taken together, although heterogeneous, the results of Marissen et al. [54], Izurieta Hidalgo et al. [13], and Schneider et al. [55] suggest that patients with acute and remitted BPD might show some form of negativity bias in their P3/LPP response to emotional stimuli.

Many studies on emotion processing in BPD have been conducted exclusively with female participants [10, 11, 13, 54–56], although the lifetime prevalence of BPD does not differ between sexes [3, 57]. Generally, sex-related differences are important to consider in brain structure and function [58], but they are widely unexplored in BPD [59]. Among healthy adults, women have an advantage in facial emotion recognition [60–63]; fMRI studies have linked this to hemispheric differences in amygdala functioning between sexes [58]. Moreover, concerning the P3/LPP, women show larger overall amplitudes [64], and there is some controversy regarding whether there is a sex-related negativity bias in the P3/LPP such that women produce larger responses than men to stimuli with negative valence [65–68]. These patterns, however, have been poorly investigated in the context of BPD-related emotion processing.

Therefore, the aim of this work was to study behavioral and neurophysiological (P3/LPP) correlates of emotional face processing in a mixed-sex sample of individuals with BPD and healthy controls. To this end, we applied the emotion classification task from our previous women-only study [13] to a male sample comprising individuals with BPD and healthy controls; the data of this men-only sample were combined with the sample from Izurieta Hidalgo et al. [13]. We hypothesized that compared with healthy controls, individuals with BPD would show a negativity bias in their behavioral ratings, while eliciting smaller P3/LPP responses to (especially predominantly happy) emotional faces. Regarding the P3/LPP, we expected to replicate the earlier finding that women would produce larger overall P3/LPP waves than men. Our further, exploratory interest was to see whether BPD diagnosis and sex would interact in the behavioral and/or neurophysiological responses to emotional faces.

With few exceptions, P3/LPP studies in individuals with BPD and in healthy controls have been based on scalp-level analyses, i.e., responses are analyzed at selected EEG electrodes and not at the level of their intracerebral generators. This is problematic because scalp waves are prone to mixing, smearing, and volume conduction [69, 70]. To overcome this issue, our study used spatio-temporal source analysis [71, 72] where current sources are modeled to represent the generators of the scalp-measured EEG; the resulting source model includes the spatial position of each source and its activity over time. Source modeling is a well-established analysis technique for neurophysiological data [69, 70, 73, 74] and has been used to assess, e.g., evoked responses in schizophrenia research [75–77], or cortical auditory fields in healthy individuals [78, 79]. Specifically, an early study of Hegerl and Frodl-Bauch [80] demonstrated that source modeling is appropriate to investigate the cerebral origins of the P3/LPP. Regarding the present study, we applied spatio-temporal source analysis to the mixed-sex dataset and expected that the P3/LPP patterns reported previously regarding group, sex, and facial emotion would be clearly visible in the intracortical source activity.

## Materials and methods

### Participants

We recruited 62 medication-free individuals with a current BPD diagnosis (DSM-IV; [81]) and 49 healthy control (HC) participants. Recruitment was done centrally within the KFO 256, a Clinical Research Unit funded by the German Research Foundation dedicated to investigating mechanisms of disturbed emotion processing in BPD [17]. All projects from the KFO 256 include subjects from a joint database. The sensor-level EEG and behavioral data of the female sample were reported previously [13].

Exclusion criteria were neurological disorders, severe medical illness, psychotropic medication for two weeks before participation, lifetime diagnosis of schizophrenia, schizoaffective or bipolar disorder, alcohol/drug abuse within the last two months, and alcohol/drug dependence within the last 12 months. HC participants had never received a psychiatric diagnosis or undergone psychological or psychiatric treatment. Data of eight BPD (4 females, 4 males) and one female HC participant were excluded from analyses due to positive toxicology screenings (*N* = 6), organic brain damage (*N* = 2), and technical malfunctioning (*N* = 1); further, EEG data of one female BPD and two female HC participants were excluded because of insufficient trial numbers due to uncorrectable artifacts (*N* = 2) or corrupt data files (*N* = 1). Therefore, the behavioral results were based on data from 54 BPD (33 females, 21 males) and 48 HC (28 females, 20 males) participants; EEG results were based on data from 53 BPD (32 females, 21 males) and 46 HC (26 females, 20 males) participants. According to power analysis (GPower 3.1; [82]), this sample size is large enough to detect moderate group by sex interaction effects (η^2^ ≥ 0.06) with 1-ß ≥ 0.80 and α ≥ 0.05.

### Clinical diagnostics

For all participants, the diagnostic process comprised an extensive telephone screening for inclusion/exclusion criteria (approx. 45 min) followed by an on-site diagnostic appointment (approx. 3 h), including a structured clinical interview with a trained physician or psychologist.

Table 1 summarizes the demographic and psychometric data. Participant groups were matched for age and intelligence; the Raven’s progressive matrices test [83] was used as an estimate for intelligence. BPD diagnosis and axis-II comorbidities were assessed with the International Personality Disorder Examination (IPDE; [84]). Axis-I comorbidities were assessed with the Structured Clinical Interview for DSM-IV (SCID-I; [85]; for comorbidities, cf. Table 2). BPD symptom severity was assessed with the Borderline Symptom List (BSL; [86]), state and trait anger with the State–Trait-Anger-Expression Inventory (STAXI; [87]), emotion dysregulation with the Difficulties in Emotion Regulation Scale (DERS; [88]), and depressiveness with the Beck Depression Inventory (BDI-II; [89]).

**Table 1:** Demographic and psychometric overview

	BPD (*N* = 54)	HC (*N* = 48)	*p*
Age (years)	27.4 (7.0)	27.6 (6.3)	0.838
Intelligence score	109.7 (11.3)	111.7 (12.2)	0.555
Number of DSM-IV BPD criteria	6.4 (1.4)	0 (0.2)	< 0.001***
Borderline symptomatology	2.1 (0.6)	0.1 (0.2)	< 0.001***
Anger	25.2 (6.9)	14.0 (3.3)	< 0.001***
Depressiveness	27.5 (9.2)	3.5 (2.9)	< 0.001***
Difficulties in Emotion Regulation	117.4 (17.9)	62.1 (13.3)	< 0.001***

Data (means and standard deviations) are shown for individuals with borderline personality disorder (BPD) and healthy controls (HC)

**Table 2:** Comorbidities of individuals with borderline personality disorder

Comorbidity	Current (%)	Lifetime (%)
Major depression	13 (24.1)	36 (66.7)
Dysthymia	8 (14.8)	n/a
Anxiety disorders	22 (40.7)	6 (11.1)
Posttraumatic stress disorder	19 (35.2)	2 (3.7)
Eating disorders	20 (37.0)	8 (14.8)
Substance use disorders^1^	4 (7.4)	10 (18.5)
Somatoform disorders	6 (11.11)	n/a
Avoidant personality disorder	14 (25.9)	2 (3.7)
Antisocial personality disorder	2 (3.7)	2 (3.7)

^1^*N* = 4 current nicotine abuse, *N* = 10 remitted substance use disorders

### Emotion classification paradigm

The emotion classification paradigm consisted of a validated forced-choice task [8, 90–93] in which participants classified faces depicting blends of angry and happy expressions by pressing a corresponding key for “angry” or “happy”, as quick as possible. Facial stimuli included female and male faces [94] depicting happy and angry expression; they were morphed in 10% steps, resulting in seven blends of angry and happy expressions (anger%:happiness%): A20:H80, A30:H70, A40:H60, A50:H50, A60:H40, A70:H30, A80:H20. Faces will be referred to as happy faces (A20:H80, A30:H70, A40:H60), ambiguous faces (A50:H50) and angry faces (A60:H40, A70:H30, A80:H20) in the remainder of this paper. Half of the faces within each emotional blend were preceded by a happily and half by an angrily intonated sentence. Each trial started with the presentation of a fixation cross at the center of the screen (100 ms) followed by an auditory stimulus (2000 ms), another fixation cross (100 ms), the emotional face (response-locked presentation, approx. 1100 ms), and a third fixation cross (450–650 ms). In total, the experiment comprised three runs á 280 trials; the total participation time was about 90 min, including preparation, training and breaks.

### EEG data acquisition and processing

During the experimental task, the EEG was simultaneously recorded with silver/silver chloride electrodes from 60 sites (equidistant reference system, Easy-Cap GmbH, Herrsching, Germany), using an average reference. Additionally, vertical and horizontal eye movements were recorded through electrodes at the epicanthus of each eye and from the supra- and infra-orbital positions of the left eye. Impedances were below 10 kΩ and signals were amplified with a QuickAmp amplifier (Brain Products GmbH, Gilching, Germany). The pass-band was set to 0.01–200 Hz, with a sampling rate of 1000 Hz. Data pre-processing was conducted with Brain Vision Analyzer 2.0 (Brain Products) and included relabeling of EEG channels according to the 10/20 system [95], digital filtering (0.1–40 Hz), correction of eye-movement artifacts [96], semiautomatic rejection of trials with non-physiological artifacts, segmentation into 1100 ms epochs (−100 to 1000 ms around stimulus onset) and baseline correction (reference: − 100 to 0 ms relative to stimulus onset). Separate averages were computed, individually for each participant and for each electrode.

Subsequently, data from the whole mixed-sex sample were subjected to BESA 5.2 (BESA GmbH, Gräfelfing, Germany) for spatio-temporal source analysis [71, 72]. We fitted a model with two symmetric regional sources (one in each hemisphere; Talairach coordinates (mm): *x* =  ± 40.3, *y* = − 80.1, *z* = 8.7; [97]), based on the EEG data pooled across all participants and experimental conditions, and with a narrow fitting window centered around the P3/LPP peak. The model was then applied as spatial filter to derive the individual source waves; here, source locations were fixed and orientations were rotated to catch maximum activity within the first spatial trace of each regional source, separately for each participant and condition. Additionally, a principal component analysis was calculated over the last few milliseconds of every epoch to compensate for drift [98].

### Statistical analyses

Statistical analyses were performed in IBM SPSS Statistics (IBM, Corp., Armonk, USA) using repeated measure analyses of variance (rmANOVAs); the Huynh–Feldt procedure [99] was applied where necessary to correct for violations of the sphericity assumption. Preliminary analyses did not reveal interactions of voice intonation or sex of the depicted faces with the between-subject factors (all *p*’s > 0.10); therefore, data were pooled accordingly to increase statistical power.

**Fig. 1:**
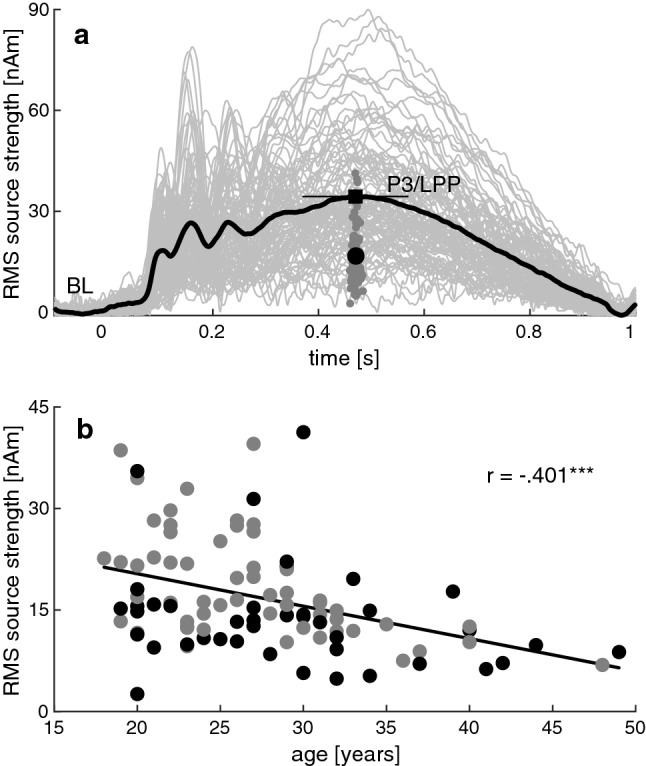
Derivation of P3/LPP magnitude measures and its covariation with age. The upper panel **a** shows the RMS source waveforms of all participants (light gray), pooled across conditions and hemispheres, and the corresponding grand-average waveform (bold black). The P3/LPP peak is marked by a black square, together with a 200-ms interval (thin black line) in which the waveform centroids in the P3/LPP peak region were calculated. The centroids of the single participants and of the grand-average waveform are denoted with small dark gray and large black circles, respectively. *BL* baseline. The lower panel **b** of the figure depicts the relation between P3/LPP magnitude and the age of male (black) and female (gray) participants

Regarding the behavioral data, rmANOVAs were conducted separately for emotion classification (calculated as the percentage of trials in which participants pressed the “angry” button when classifying a face) and response time (log-transformed to handle skewness); EEG-related rmANOVAs were based on the P3/LPP magnitude in a 200-ms interval centered around its peak, expressed as the *y*-value of the centroid in the aggregate root-mean-square (RMS) waveform of all three regional source orientations (Fig. 1a). All rmANOVAs included the factors GROUP (BPD vs. HC), SEX (males vs. females) and COND (7 levels of anger:happiness proportion); EEG analyses also included a factor HEMISPHERE (left vs. right hemisphere). To assess the role of emotional valence and ambiguity in detail, we also performed separate rmANOVAs in which the fully ambiguous condition (A50:H50) was omitted and the CONDITION factor was replaced with the two factors EMOTION (happy vs. angry emotion) and AMBIGUITY (high vs. medium vs. low ambiguity).

Age was controlled as a covariate of no-interest in all analyses because the corresponding sex difference showed a trend to significance (*t*(100) = 1.944, *p* = 0.055) and the P3/LPP magnitude is known to decrease with age [100, 101], which was also seen in our study (*r* = − 0.401, *p* < 0.001***; Fig. 1b).

## Results

### Emotion classification

**Fig. 2:**
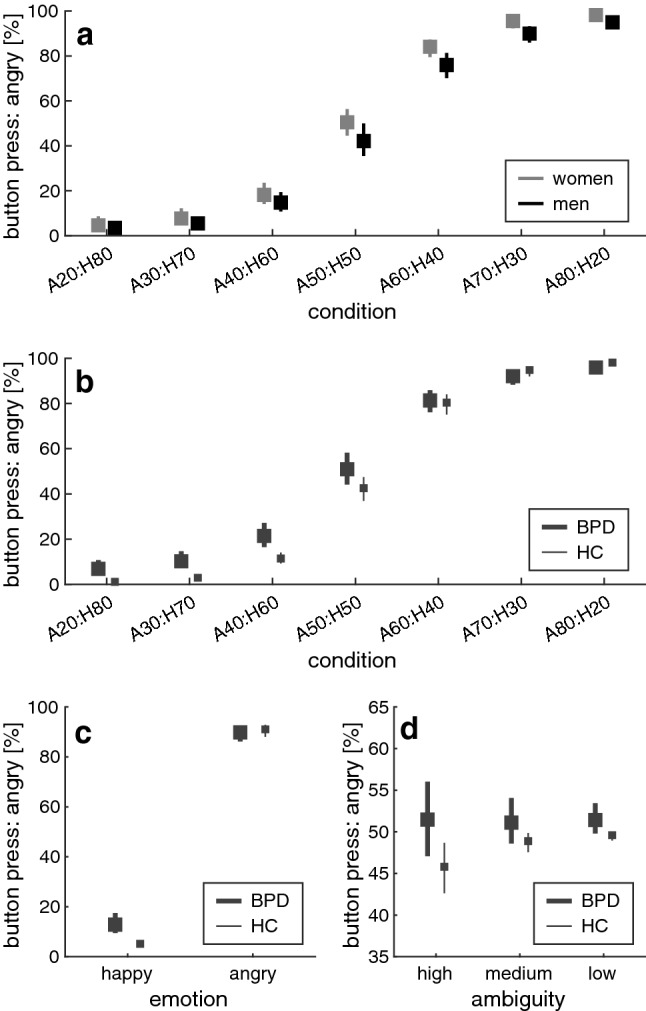
Behavioral responses in the emotion classification task. The panels depict mean proportions (%) of anger classification in **a** male vs. female participants and in **b** individuals with BPD vs. healthy controls (HC). The bottom panels show how the anger ratings of the BPD and HC groups vary with **c** the emotional valence and **d** the ambiguity of the faces presented in the task. Error bars are 95% bias-corrected and accelerated confidence intervals [102], based on 2000 resamples

Figure 2 depicts the mean responses in the emotion classification task. Participants classified faces with high proportions of anger as “angry” and faces with high proportions of happiness as “happy” (CONDITION: *F*(6,582) = 82.426, *p* < 0.001***, *ƞ*^2^ = 0.459). Furthermore, women had higher anger ratings than men, as indicated by a main effect of SEX (*F*(1,97) = 5.693, *p* = 0.019*, *ƞ*^2^ = 0.055; Fig. 2a). While the main effect of GROUP missed significance (*F*(1,97) = 3.791, *p* = 0.054, *ƞ*^2^ = 0.038), a GROUP*CONDITION interaction effect (*F*(6,582) = 6.537, *p* = 0.001**, *ƞ*^2^ = 0.063) suggested that individuals with BPD showed a higher frequency of anger ratings than healthy controls for predominantly happy and ambiguous faces (Fig. 2b). There was no GROUP*SEX interaction (*F*(1,97) = 0.112, *p* = 0.739, *ƞ*^2^ = 0.001).

In the second analysis step, the fully ambiguous condition (A50:H50) was removed and the CONDITION factor was substituted with the factors EMOTION and AMBIGUITY. Again, there was a main effect of SEX (*F*(1,97) = 5.724, *p* = 0.019*, *ƞ*^2^ = 0.056), as described above, and a main effect of EMOTION (*F*(1,97) = 212.503, *p* < 0.001***, *ƞ*^2^ = 0.687) occurred together with an EMOTION*AMBIGUITY interaction (*F*(2,194) = 29.257, *p* < 0.001***, *ƞ*^2^ = 0.232). The GROUP factor was found to interact with EMOTION (*F*(1,97) = 12.114, *p* = 0.001**, *ƞ*^2^ = 0.111) and AMBIGUITY (*F*(2,194) = 4.102, *p* = 0.039*, *ƞ*^2^ = 0.041). This indicates that compared with HC participants, individuals with BPD showed higher frequencies of anger ratings in response to happy but not angry faces (Fig. 2c), and that the difference in the anger ratings of both groups was pronounced in faces with higher emotional ambiguity (Fig. 2d). There was no GROUP*SEX interaction (*F*(1,97) = 0.000, *p* = 0.991, *ƞ*^2^ = 0.000), nor were there other significant effects in participants’ emotion classifications.

### Response times

**Fig. 3:**
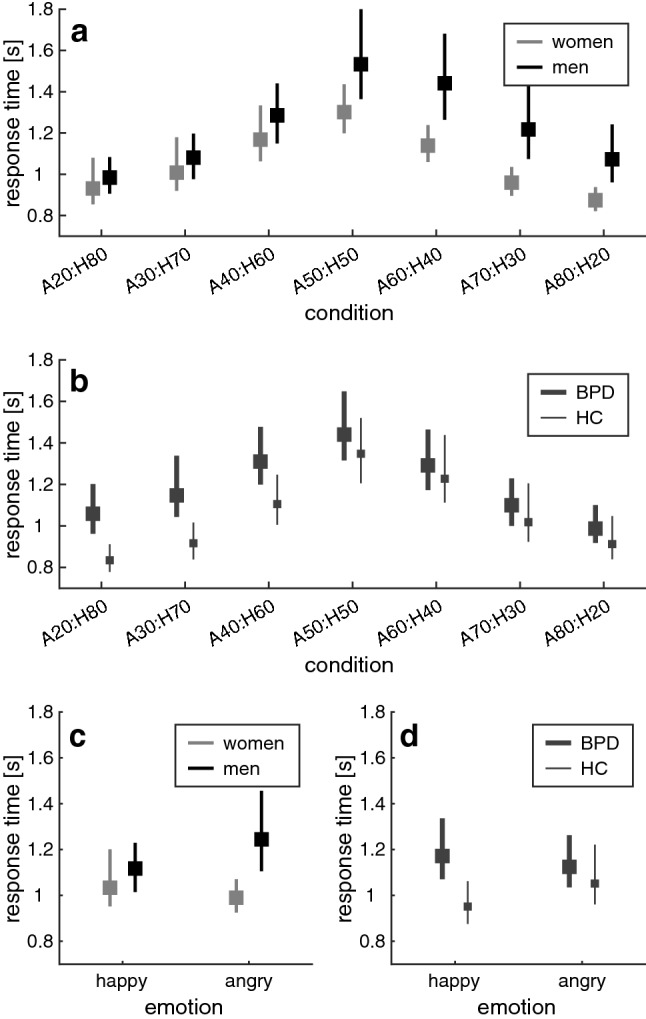
Response times in the emotion classification task. The panels depict mean response times in **a** male vs. female participants and in **b** individuals with BPD vs. healthy controls (HC). The bottom panels show how the response times in conditions with different emotional valence vary in **c** male vs. female participants and in **d** the BPD vs. the HC group. Error bars are 95% bias-corrected and accelerated confidence intervals [102], based on 2000 resamples

Figure 3 presents the mean response times in the emotion classification task. Participants generally responded faster to faces with lower emotional ambiguity (CONDITION: *F*(6,582) = 6.164, *p* = 0.001**, *ƞ*^2^ = 0.060). Men responded slower than women (SEX: *F*(1,97) = 5.598, *p* = 0.020*, *ƞ*^2^ = 0.055), especially to ambiguous faces and faces with high proportions of anger (SEX*CONDITION: *F*(6,582) = 3.015, *p* = 0.042*, *ƞ*^2^ = 0.03; Fig. 3a). Individuals with BPD responded slower than healthy controls (GROUP: *F*(1,97) = 4.788, *p* = 0.031*, *ƞ*^2^ = 0.047), particularly to faces with high proportions of happiness (GROUP*CONDITION: *F*(6,582) = 4.536, *p* = 0.008**, *ƞ*^2^ = 0.045; Fig. 3b). There was no GROUP*SEX interaction (*F*(1,97) = 0.357, *p* = 0.551, *ƞ*^2^ = 0.004).

When the ambiguous condition (A50:H50) was omitted and the CONDITION factor was replaced with EMOTION and AMBIGUITY, the main effects of GROUP (*F*(1,97) = 5.670, *p* = 0.019*, *ƞ*^2^ = 0.055) and SEX (*F*(1,97) = 5.866, *p* = 0.017*, *ƞ*^2^ = 0.057) were present as reported above, together with AMBIGUITY (*F*(2,194) = 11.055, *p* < 0.001***, *ƞ*^2^ = 0.102). While the EMOTION factor missed significance as a main effect (*F*(1,97) = 3.530, *p* = 0.063, *ƞ*^2^ = 0.035), it was found to interact with both between-subject variables (SEX*EMOTION: *F*(1,97) = 4.946, *p* = 0.028*, *ƞ*^2^ = 0.049; GROUP*EMOTION: *F*(1,97) = 6.344, *p* = 0.013*, *ƞ*^2^ = 0.061): Men responded slower than women to angry but not happy faces (Fig. 3c); in turn, individuals with BPD responded slower than healthy controls to happy but not to angry faces (Fig. 3d). There was no GROUP*SEX interaction (*F*(1,97) = 0.364, *p* = 0.548, *ƞ*^2^ = 0.004), nor were there other significant effects in participants’ response times.

### P3/LPP activity

**Fig. 4:**
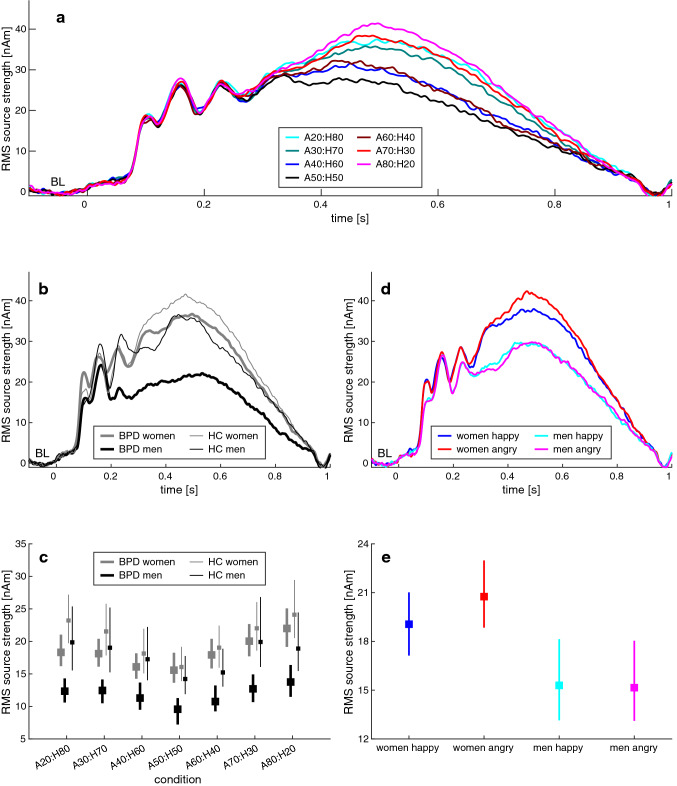
P3/LPP activity in response to the emotional faces from the classification paradigm. The figure shows how the P3/LPP wave varies according to the main effects of **a** condition and **b** sex and group. The bottom left panel **c** presents the P3/LPP magnitude in the different experimental conditions. The lower right panels of the figure (**d**, **e**) depict the impact of emotional valence on the P3/LPP magnitude in male vs. female participants. *BPD* individuals with borderline personality disorder, *HC* healthy controls, *BL* baseline. The 95% confidence intervals in the bottom panels of the figure are bias-corrected and accelerated as suggested by DiCiccio and Efron [102], based on 2000 resamples

Figure 4 presents the P3/LPP responses from the emotion classification task. There was a significant main effect of COND (*F*(6,564) = 5.032, *p* < 0.001***, *ƞ*^2^ = 0.051; cf. Fig. 4a), suggesting that the P3/LPP magnitude varied with the emotional expression of the faces. Figure 4b illustrates the effects of the participant´s characteristics: Women had larger responses than men (SEX: *F*(1,94) = 6.844, *p* = 0.010*, *ƞ*^2^ = 0.068), and individuals with BPD produced smaller responses than healthy controls (GROUP: *F*(1,94) = 9.961, *p* = 0.002**, *ƞ*^2^ = 0.096). There was no GROUP*SEX interaction (*F*(1,94) = 2.585, *p* = 0.111, *ƞ*^2^ = 0.027).

Figure 4c presents the interplay of emotional faces and P3/LPP magnitude in more detail, for the BPD and HC group, and separately according to sex. The magnitude variance in the different conditions was stronger in female than in male participants (SEX*CONDITION: *F*(6,564) = 2.471, *p* = 0.033*, *ƞ*^2^ = 0.026), and it was also stronger in healthy controls than in individuals with BPD (GROUP*CONDITION: *F*(6,564) = 2.765, *p* = 0.019*, *ƞ*^2^ = 0.029).

As with the behavioral data, P3/LPP responses were also analyzed without the neutral condition (A50:H50), considering the factors EMOTION and AMBIGUITY instead of CONDITION. Again, there were main effects of both participant characteristics (SEX: *F*(1,94) = 6.485, *p* = 0.013*, *ƞ*^2^ = 0.065; GROUP: *F*(1,94) = 10.162, *p* = 0.002**, *ƞ*^2^ = 0.098), and a main effect of AMBIGUITY indicated that the P3/LPP increased in magnitude when faces turned less ambiguous (*F*(2,188) = 7.819, *p* = 0.001**, *ƞ*^2^ = 0.077). Women showed a larger P3/LPP magnitude difference than men in response to faces with angry vs. happy valence (SEX*EMOTION: *F*(1,94) = 5.580, *p* = 0.020*, *ƞ*^2^ = 0.056; Fig. 4d and 4e); however, the interactions of emotional ambiguity with both participant characteristics as well as the interaction of group with emotional valence missed significance (GROUP*AMBIGUITY: *F*(2,188) = 2.990, *p* = 0.057, *ƞ*^2^ = 0.031; SEX*AMBIGUITY: *F*(2,188) = 2.502, *p* = 0.089, *ƞ*^2^ = 0.026; GROUP*EMOTION: *F*(1,94) = 3.487, *p* = 0.065, *ƞ*^2^ = 0.036). There was no GROUP*SEX interaction (*F*(1,94) = 2.272, *p* = 0.135, *ƞ*^2^ = 0.024), nor were there other significant P3/LPP effects.

## Discussion

This study investigated behavioral and source-level neurophysiological correlates (P3/LPP) of emotional face processing in a mixed-sex sample of individuals with BPD and healthy volunteers. Our data show that BPD diagnosis and participant´s sex influence emotion processing at the face categorization stage. In the following, we discuss our findings in the context of existing evidence and elaborate on some limitations and implications for future research.

Individuals with BPD generally responded slower than healthy controls to emotional faces, especially to those with high proportion of happiness; furthermore, they showed more anger ratings in happy faces, and in faces with high emotional ambiguity. This pattern indicates that individuals with BPD have a negativity bias in their categorization of facial emotion, corroborating our hypothesis and previous findings [8, 10]. The present study also shows a P3/LPP magnitude decrement in male individuals with BPD, which resembles findings in women with acute and remitted BPD [13, 55], and the P3/LPP variability in response to the different facial expressions appeared smaller in the BPD group than in the control group. The latter effect, however, was only observed during primary analysis and shortly missed significance when data were analyzed with respect to emotional valence and ambiguity. Therefore, while BPD-related changes in cortical emotion processing are likely, one should resist the temptation to draw too specific conclusions from the current data. Beyond group comparisons, the P3/LPP in our study closely mirrored facial emotion ambiguity, in line with earlier research [39, 41–43], but there was no negativity bias in the sense that angry faces would elicit larger P3/LPP responses than happy faces [36, 44, 45]. Hajcak and Foti [40] have proposed that the P3/LPP represents stimulus ‘significance’; with this in mind, the overall P3/LPP decrement in individuals with BPD might point to a more general difficulty in cortical categorical emotion processing. To date, our work is one of very few that addresses P3/LPP alterations in BPD at all; we hope that our results will stimulate future investigations to assess the interplay of emotional valence and intensity in the P3/LPP in more detail, in individuals with BPD and also in healthy individuals.

In the current study, female participants showed increased anger ratings in response to the emotional faces, and they responded faster than males, especially in faces with ambiguous or angry expression. On the level of neural processing, this was accompanied by enhanced P3/LPP waves, in line with our hypothesis and the literature [64]; moreover, women but not men showed larger P3/LPP responses to angry vs. happy faces. These results are compatible with the idea of an ‘advantage’, but also a negativity bias in female processing of facial emotions [65, 66]. The sex-related response time differences might alternatively be understood in terms of a male ‘positivity bias’, in the sense that males need longer to categorize faces with angry expression; however, we do not know earlier findings that would support such an interpretation, and it is also not reflected in the P3/LPP. Apparently, sex did not seem to interact with BPD diagnosis in any behavioral or P3/LPP-related measure. This is interesting given that many studies on emotion processing in BPD have been based on female-only samples, although lifetime prevalence is comparable between sexes [57]; and one might now conclude that there is no interplay of both characteristics at the stage of behavioral and neurophysiological facial emotion categorization. It is, however, important to acknowledge that despite its substantial sample size, our experiment might have been underpowered to assuredly exclude the presence of small group by sex interactions, at least regarding P3/LPP (cf. Fig. 4d). The current results should, therefore, be replicated based on a larger sample, particularly since sex is also known to influence other aspects of emotion dysregulation in BPD (e.g., aggression; [24]).

Contrary to most previous work, the present experiment employed spatio-temporal source analysis [71, 72, 80] to study cortical correlates of emotional face processing. Our modeling included a grand-average fit with symmetric regional sources and a centroid measure of response magnitude that could be reliably determined in each participant and condition. Despite these robust parameters, the results demonstrate that source modeling is well suited to analyze the intracerebral P3/LPP generators and catch effects of participant characteristics and stimulus features on this wave; in fact, future experiments might be able to uncover even more subtle differences by allowing individualized source localization in their paradigms.

The current study incorporated a large, well-balanced, mixed-sex sample of medication-free individuals with BPD and healthy controls that was carefully matched for age and intelligence. It should, however, be noted that the lack of a clinical control group renders it challenging to draw illness-specific conclusions in a disorder like BPD that is known to have a typical pattern of comorbidities [103]. This is of particular importance since the rate of comorbidities was high in the BPD group which is consistent with earlier reports (e.g., [103]) and thus supports the representativeness of the current sample, but questions the specificity of the current results for BPD. As mentioned above, a replication in a large mixed-sex sample including clinical control groups is needed. Furthermore, the emotion classification task in our experiment was restricted to blends of happy and angry faces and did not include a measure of participant’s arousal during the task. Given the interpretation of the P3/LPP as a process that reflects stimulus significance [36], future studies should explicitly include such a measure in their design and extend the scope of analysis to other facial emotions like, e.g., fear or disgust.

Notwithstanding these limiting factors, the present work has shown that BPD-related alterations in behavioral and neurophysiological correlates of facial emotion processing exist in both male and female individuals, and that spatio-temporal source analysis is a valuable tool to access the corresponding intracortical processes (P3/LPP) at the categorization stage. We hope that this study will deepen our knowledge about the psychological and neural underpinnings of BPD in a way that, on the long run, may help to improve therapeutic approaches to emotional dysregulation in this severe illness.
